# Comparative study on the customization of natural language interfaces to databases

**DOI:** 10.1186/s40064-016-2164-y

**Published:** 2016-04-30

**Authors:** Rodolfo A. Pazos R., Marco A. Aguirre L., Juan J. González B., José A. Martínez F., Joaquín Pérez O., Andrés A. Verástegui O.

**Affiliations:** División de Estudios de Posgrado e Investigación, Tecnológico Nacional de México/Instituto Tecnológico de Ciudad Madero, Av. 1o. de Mayo s/No., Col. Los Mangos, Ciudad Madero, Tamaulipas Mexico; Departamento de Ciencias Computacionales, Tecnológico Nacional de México/Centro Nacional de Investigación y Desarrollo Tecnológico, Interior Internado Palmira S/N, Col. Palmira, Cuernavaca, Morelos Mexico

**Keywords:** Natural language processing, Natural language interface, Databases, Semantic modelling

## Abstract

In the last decades the popularity of natural language interfaces to databases (NLIDBs) has increased, because in many cases information obtained from them is used for making important business decisions. Unfortunately, the complexity of their customization by database administrators make them difficult to use. In order for a NLIDB to obtain a high percentage of correctly translated queries, it is necessary that it is correctly customized for the database to be queried. In most cases the performance reported in NLIDB literature is the highest possible; i.e., the performance obtained when the interfaces were customized by the implementers. However, for end users it is more important the performance that the interface can yield when the NLIDB is customized by someone different from the implementers. Unfortunately, there exist very few articles that report NLIDB performance when the NLIDBs are not customized by the implementers. This article presents a semantically-enriched data dictionary (which permits solving many of the problems that occur when translating from natural language to SQL) and an experiment in which two groups of undergraduate students customized our NLIDB and English language frontend (ELF), considered one of the best available commercial NLIDBs. The experimental results show that, when customized by the first group, our NLIDB obtained a 44.69 % of correctly answered queries and ELF 11.83 % for the ATIS database, and when customized by the second group, our NLIDB attained 77.05 % and ELF 13.48 %. The performance attained by our NLIDB, when customized by ourselves was 90 %.

## Introduction

Databases (DBs) are present everywhere, many applications access them at all times, and in many cases information obtained from them is used for making important business decisions. Obtaining unusual or complex information from a database demands from users to have broad knowledge of a DB query language such as SQL. Unfortunately, most users (for example, high-ranking managers) do not have the necessary expertise for formulating queries for obtaining such information.

NLIDBs are important tools since they allow users accessing information in a database by a query formulated in natural language (NL). The purpose of these systems is to facilitate the querying task to users by sparing them the burden of having to learn a DB query language. With a NLIDB, a user simply types a query in natural language, similarly as he/she would do when communicating with another person, and the interface interprets the query and translates it to a DB query language statement, which is submitted by the NLIDB to a DB management system to get the information requested.

There exists another type of systems similar to NLIDBs, known as Question and Answering (Q&A) systems. Unlike these, NLIDBs return a result which is the output of a query submitted to a database. Additionally, Q&A systems return a set of results (which can be derived from a search in documents, texts, etc.), from which the user has to choose the one that he/she considers correct. An example of this type of Q&A systems is presented in Komiya et al. (2013).

One of the most complex problems faced by NLIDBs concerns their ability to be used for querying different databases (known as domain independence). Each time a domain independent NLIDB is required to query a new database, it is necessary to previously customize it for this particular database. To this end, it is necessary to supply the system with the words and necessary concepts for the new domain, which are related to the information stored in the database.

At this point it is convenient to emphasize that the success rate of NLIDBs highly depends on the quality of their customization. Additionally, it is important to take into consideration that, for domain independent NLIDBs, it is difficult to obtain a success rate (percentage of correctly translated queries) higher than 90 %. Therefore, the importance of carrying out the best customization possible in order to obtain the maximum success rate that a NLIDB can achieve. Normally, the customization process for adapting a NLIDB to a specific database requires a high level of expertise from DB administrators and spending long times in the customization process, which sometimes is carried out by trial and error.

Despite the fact that NLIDBs have been implemented since the 60s, their performance (success rate) has been disappointing, since there does not exist a domain independent NLIDB that achieves a success rate (recall) close to 100 %. An indication of the complexity of the issues present in the translation process is revealed by the current situation of commercial NLIDBs: LanguageAccess (developed by IBM), English Query (commercialized by Microsoft) and DataTalker (developed by Natural Language Inc.), among others, have been discontinued.

In this article we present results from an experiment aimed at comparing the effectiveness and easiness of customization of ELF (Elf 2015) and our NLIDB, which is based on a new semantically-enriched data model described in Pazos et al. (2011) and a layered architecture for the translation of NL queries to SQL. The data dictionary (called semantic information dictionary) of our interface permits to carry out a customization, which consists of: relating words or phrases from different syntactic categories (such as nouns, verbs, adjectives and prepositions) to DB tables and columns, as well as defining imprecise values (those that represent a value range, such as morning, afternoon, evening) as equivalent to value ranges, and defining aliases (those values that can be denoted in different ways, such as: noon, equivalent to 12:00 hrs.; midnight, equivalent to 0:00 hrs.; dozen, equivalent to 12) as equivalent to specific values. This functionality permits to solve problems not previously addressed by other NLIDBs.

## Background

The first NLIDBs were developed in the 60s and were basically natural language interfaces for expert systems, which were custom made for a specific domain. Most of those NLIDBs were developed having in mind a particular database. Consequently, they could not be easily modified for querying a different database and it was impossible to port them to other application domains. These systems had a knowledge base manually created by domain experts, which was tightly interwoven with the interface source code.

Currently, NLIBDs that offer portability to different databases use techniques for achieving some semi-customization of the domain dictionary, such as automatically obtaining information of the DB structure from the DB schema, adding synonyms to the descriptions of DB tables and columns, and obtaining information from pairs of NL queries and SQL statements.

Easiness of customization is a very desirable feature in NLIDBs, and it should be provided in such a way that a DB administrator could be able to customize the interface without a deep knowledge of the inner workings of the system in order to achieve the highest success rate possible for the interface.

Of the vast amount of the literature published on NLIDBs, only a small proportion deals with NLIDB customization, and from this, just a few articles present performance results where the NLIDBs are customized by users different from the implementers. The rest of this section describes the most recent and relevant works on NLIDB customization.

Masque/sql (Androutsopoulos et al. 1993) is an interface that performs a semiautomatic customization process. Unlike previously developed interfaces, Masque/sql has a simple domain editor for helping users to relate words to concepts of the DB schema and to define the meaning of each word in terms of logical predicates. The interface was tested on a geographical database from a real-life application, for which the customization process consisted of relating 86 words to the elements of the DB schema (tables and columns). Unfortunately, no quantitative results are reported on the performance of Masque/sql.

NLIDBs such as Precise (Popescu et al. 2004a) use an automatic customization, where the names of all the DB elements are extracted and separated into words with their respective synonyms, so they can be later identified during the translation process. Additionally, the interface permits manually augmenting the lexicon with relevant synonyms, prepositions, etc. Experiments were conducted using a customization of the lexicon performed by the implementers and the databases described in Tang and Mooney (2001) and the ATIS database (Atis 2015). For the first databases Precise obtained approximately 80 % recall (and 100 % accuracy), and for ATIS the interface obtained 93.8 % accuracy (Popescu et al. 2004b); unfortunately no recall value was reported for ATIS.

The virtual library of the Concordia University uses a NLIDB called NLPQC (Stratica et al. 2005). This interface can be manually customized by the NLIDB administrator using files that contain templates with the semantic structure of the queries supported. Despite claims that NLPQC is domain independent, only tests for a library domain have been reported. The experiments reported are of the proof-of-functionality type and no accuracy or recall figures are given.

In the Clinical E-Science Framework (CLEF), which is a repository of clinical histories for biomedical research and medical care, a technique known as WYSIWYM (Hallet et al. 2007) is used, which uses a semantic graph inspired by the one described in Zhang et al. (1999). The customization was carried out by the implementers by supplying a knowledge base of the semantic part of the database (semantic graph), which permits generating automatically the components that users may choose. The experiment for that system involved 15 physicians with knowledge of the domain, which were given a 5-to-10-min briefing on the interface operation and a set of four queries. The average time that took them to formulate a query was 3.9 min and the reported recall was 100 %. Finally, it is important to mention that WYSIWYM does not permit formulating queries in unrestricted natural language, but rather the user starts by editing a basic query template, where concepts to be instantiated are clickable spans of text with associated pop-up menus containing options for expanding the query.

DaNaLIX (Li et al. 2007) is an interface for querying XML databases. The system uses transformation rules (like those used in Prolog), where query elements are compared by using parse trees. This system can be automatically customized by learning new rules from its interaction with users. The evaluation was carried out by comparing DaNaLIX, a previous version (NaLIX) and two other systems: Cocktail and GENLEX. The test was performed on two deductive databases Geobase and Jobdata with corpora consisting of 880 and 640 queries respectively (Tang and Mooney 2001). In the experiment the customization consisted of extracting rules from the training corpora for each domain, afterwards the rules were loaded into NaLIX, and the results of the translation process are XQuery statements (a query language for XML). NaLIX attained 81 % recall for both corpora.

One of the current NLIDBs that has a large customization functionality is C-Phrase (Minock 2010). It has an authoring tool for editing the interface data dictionary for a specific domain, by which it can relate words to DB tables, columns and join paths of the DB schema. It also permits to edit rules and patterns supported by the interface and substitutions of phrases by concepts that the interface can interpret. The evaluation of C-Phrase was carried out on the Geoquery database described in Tang and Mooney (2001), and it was customized by several undergraduate students, which were given a user manual of the interface and a training set of 100 randomly selected queries for customizing the interface in 2 h. C-Phrase attained approximately 75 % recall.

In Giordani and Moschitti (2009) a work is presented that focuses on the use of machine learning algorithms for automatically mapping NL queries to SQL statements. Their approach consists of selecting SQL statements for NL queries, where the selection of the correct SQL statement is modeled as a ranking problem using Support Vector Machines and several kernels. For training their system the Geoquery250 and the Restquery250 corpora were used, where after the generalization of queries, pairs of (NL query)–(SQL statement) were generated including positive and negative examples. The results obtained with several combined kernels were approximately 76 and 85 % accuracy for Geoquery250 and Restquery250 respectively.

In their most recent work (Giordani and Moschitti 2012), they improve their approach, using an SQL generator, which exploits syntactic dependencies in the NL queries and the DB metadata. They also used advanced machine learning techniques such as kernel-based rerankers, which improve the initial list of candidates provided by the generative analyzer. For comparison with other systems, they used the Geoquery500 corpus and the Geoquery700 corpus, obtaining an f-measure of 87 % for Geoquery500 and 85 % for Geoquery700, where f-measure is the harmonic mean of accuracy and recall.

Several NLIDBs were developed for commercialization. Some of the most successful were INTELLECT (Harris 1984), Q&A (Hendrix 1986), English Wizard (Roberts 1998) and English Query (Blum 1999). However, most of them have been discontinued.

One of the few surviving commercial interfaces, reputed as having good performance, is Access ELF. The customization of ELF is automatic, and it generates a domain dictionary that relates DB tables and columns to words of different syntactic categories. The dictionary also keeps information on the relations among DB elements, and the values stored in the database together with a reference to the DB columns where the values are stored. Additionally, ELF has a domain editor where the NLIDB administrator can relate (or modify the relation of) words to DB tables and columns. In the tests presented in Conlon et al. (2004), where the interface was customized by an expert and the end users were human resource professionals, the success rate reported (presumably recall) was 70–80 %.

Table 1 summarizes important aspects of works on the customization and evaluation of NLIDBs. The table shows that in many cases NLIDBs are customized by the implementers; unfortunately, the performance results obtained this way do not show what the performance would be if the NLIDBs were customized by users (DB administrators). Only C-Phrase reports experiments where the interface was customized by undergraduate students.

**Table 1:** Related works on customization and evaluation of NLIDBs

NLIDB	Customized by	Complexity of the DB	Complexity of the query corpora	Comparison versus other NLIDBs	Performance
Masque/sql	–	–	–	–	–
Precise	The implementers	*ATIS: high*; Mooney‘s dataset Geobase, Restbase and Jobdata DBs: low	*ATIS: high*; Mooney‘s dataset: moderate	AT&T, CMU, MIT, SRI, BBN, UNISYS, MITRE, HEY (on ATIS). EQ, Mooney (on Mooney’s dataset)	Accuracy: 93.8 % (on ATIS). *Recall: 80 %*, accuracy: 100 %, (on Mooney’s dataset)
NLPQC	Presumably the DBA	Library of the Concordia	Low	–	–
CLEF	The implementers	University: low moderate	*High*	–	*Recall: 100 %*
DaNaLIX	The implementers	Geobase, Jobdata: low	Geoquery880, Jobquery640: moderate	COCKTAIL, GENLEX, NaLIX	*Recall: ≈ 81 %*
C-PHRASE	*Undergraduate students*	Geobase: low	Geoquery880: moderate	Precise, WASP, SCISSOR, Z&C	*Recall: ≈ 75 %*, accuracy: ≈ 86 %
Giordani and Moschitti (2012)	The implementers	Geobase: low	Geoquery500, Geoquery700: moderate	Precise, Krisp Model III+R, SemResp, UBL, DCS/DCS+	F1*: 87 %
ELF, Conlon et al. (2004)	An expert	High	Unknown	–	*Recall: 70-80 %*

* F1 is the harmonic mean of accuracy and recall

## Description of our NLIDB

There have been several versions of the NLIDB developed by us (all of them permit formulating queries in Spanish). The first version, described in Pazos et al. (2005) uses a pre-processor that automatically builds a data dictionary, which permits to deal with domain independence. The translation technique involves the interpretation of nouns, prepositions and conjunctions. The second version, described in Pazos et al. (2010) includes domain-independent dialogue processes, which are based on a typification of problems occurring in queries that involve most of the cases found in queries. The experience gained in the development of these versions served us to be aware of the complexity of the problems involved in NLIDBs.

The design of the new version of our NLIDB is based on the following premise: *the translation from a NL query to an SQL statement is an extremely complex problem*. The approach used in this project differs from those utilized in other NLIDBs, in which one or a few mechanisms (v.g., pattern matching, case-based reasoning, supervised learning, statistical methods) have been applied for solving the problems occurring in queries. Our analysis of query corpora (ATIS, Geoquery, Northwind) has revealed that the problems present in queries are of very diverse nature; therefore, we think that they can not be solved by one or a few heuristics. Consequently, in this project query problems were identified and classified in order to develop a specific method for solving each class of problems. Specifically, a layered architecture was devised (similar to the OSI model for communication networks), where each functionality layer deals with a different problem in the translation process. This architecture is explained in “Proposed architecture” section.

Additionally, in order to solve several problems, described in Pazos et al. (2011), in the current version of our NLIDB we have proposed an architecture for the semantic information dictionary (SID) based on a new semantically-enriched database model (SEDBM), described in Aguirre (2014), Pazos et al. (2011), which includes relevant information for the correct translation of queries. The architecture of the SID is described in “Semantic information dictionary” section.

### Semantic information dictionary

The SID (or domain dictionary) is the keystone of our NLIDB, since it stores the information necessary for the interface to correctly interpret a query. The performance of an interface highly depends on the quantity and quality of the semantic information stored in the domain dictionary; i.e., the better the information stored the better the understanding of the NL query by the NLIDB, which in turn yields a better success rate.

The SID mainly stores words and phrases that are used for referring to DB tables and columns, as well as information of the DB schema. The customization of the NLIDB for a particular database consists of populating the SID with relevant semantic information of the database. For this reason it is important that the DB administrator performs the customization task.

For many NLIDBs, the customization process is usually lengthy, complicated, and requires a deep knowledge of the inner workings of the system in order to achieve the best success rate that the interface can deliver. Therefore, recent interfaces carry out an automatic customization process, which extracts information from the names and descriptions of the DB tables and columns stored in the data dictionary of the DB management system. This automatic customization permits reducing the time and effort for customizing the NLIDB. Unfortunately, automatically customized NLIDBs yield poor success rates, so it is necessary to fine-tune the initial customization.

Initially, our NLIDB carries out an automatic customization, which permits populating its SID based on the descriptions of the DB tables and columns and the information of relations between tables, which are stored in the data dictionary of the DB management system. Additionally, the SID of our NLIDB can keep information on words and phrases that refer to tables, columns, relations between tables, imprecise values, alias values, which permits having the necessary information and facilitates query interpretation. Usually the automatic customization is not enough for obtaining a high percentage of correctly answered queries; and consequently, it is necessary that the DB administrator fine-tunes the NLIDB for supplying the information mentioned above.

**Fig. 1:**
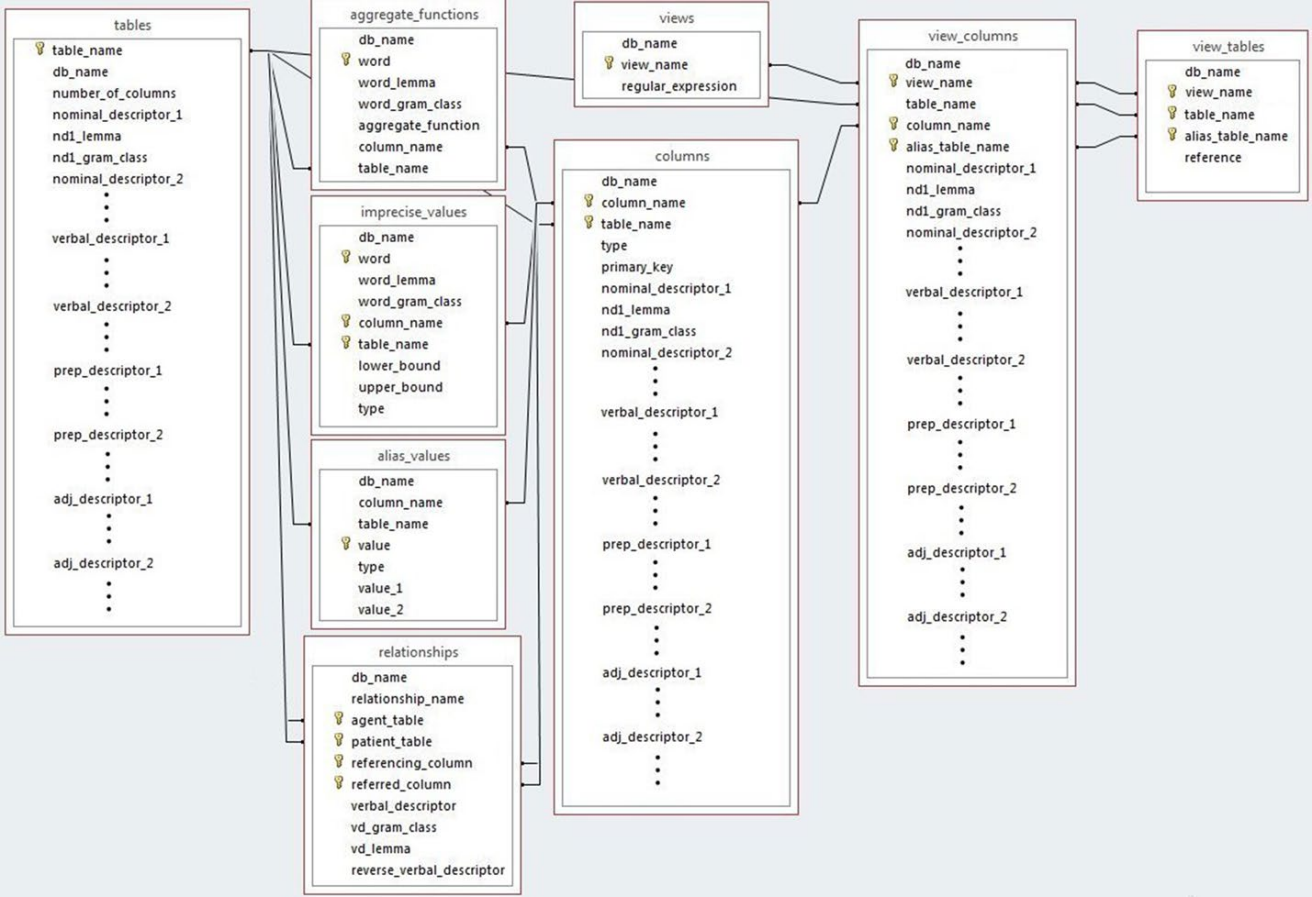
Database schema of the SID

Figure 1 presents the DB schema of the SID. The most important tables of the SID are *tables*, *columns* and *relationships*, which are used for storing information on DB tables, columns and relations between tables. These dictionary tables contain relevant information extracted from the DB schema, as well as descriptors for different syntactic categories (nouns, verbs, adjectives and prepositions) and lemmas. The SID includes three more small tables: *imprecise_values*, *alias_values* and *aggregate_functions*, which are used for storing information on imprecise values, alias values and aggregate functions. Finally, the SID includes three more tables: *views*, *view_tables* and *view_columns*, which are used for storing the definition of views [a mechanism for dealing with complex queries, whose explanation is too lengthy to be included in this article, but it can be found in Aguirre (2014)].

**Fig. 2:**
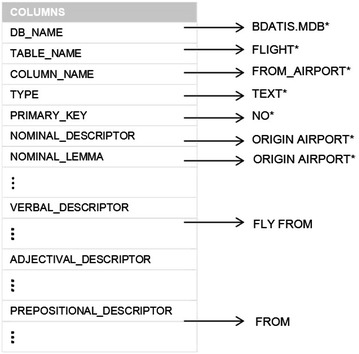
Example of column description in the SID

Figure 2 presents an example of a fragment of the SID of the relevant information that is stored for a DB column. For each DB column, the SID stores the following information: DB name, table name, column name, data type, an indication if the column participates in the primary key, 2 nominal descriptors, 2 lemmas corresponding to the preceding descriptors, 2 values indicating the success rate of the corresponding descriptor (for future use), 2 values indicating the occurrence rate of the corresponding descriptor (for future use), 2 verbal descriptors (and the corresponding lemmas, success rates and occurrence rates), 2 adjectival descriptors (and the corresponding lemmas, success rates and occurrence rates), and 2 prepositional descriptors (and the corresponding lemmas, success rates and occurrence rates).

Finally, it is worth mentioning that the size of the SID is proportional to the size of the database schema; specifically it is several times as large; and it is independent of the amount of data stored in the database (unlike other NLIDBs such as ELF).

For better understanding the customization process of the SID, the description of this process is explained in “Customization of the semantic information dictionary” section.

### Proposed architecture

**Fig. 3:**
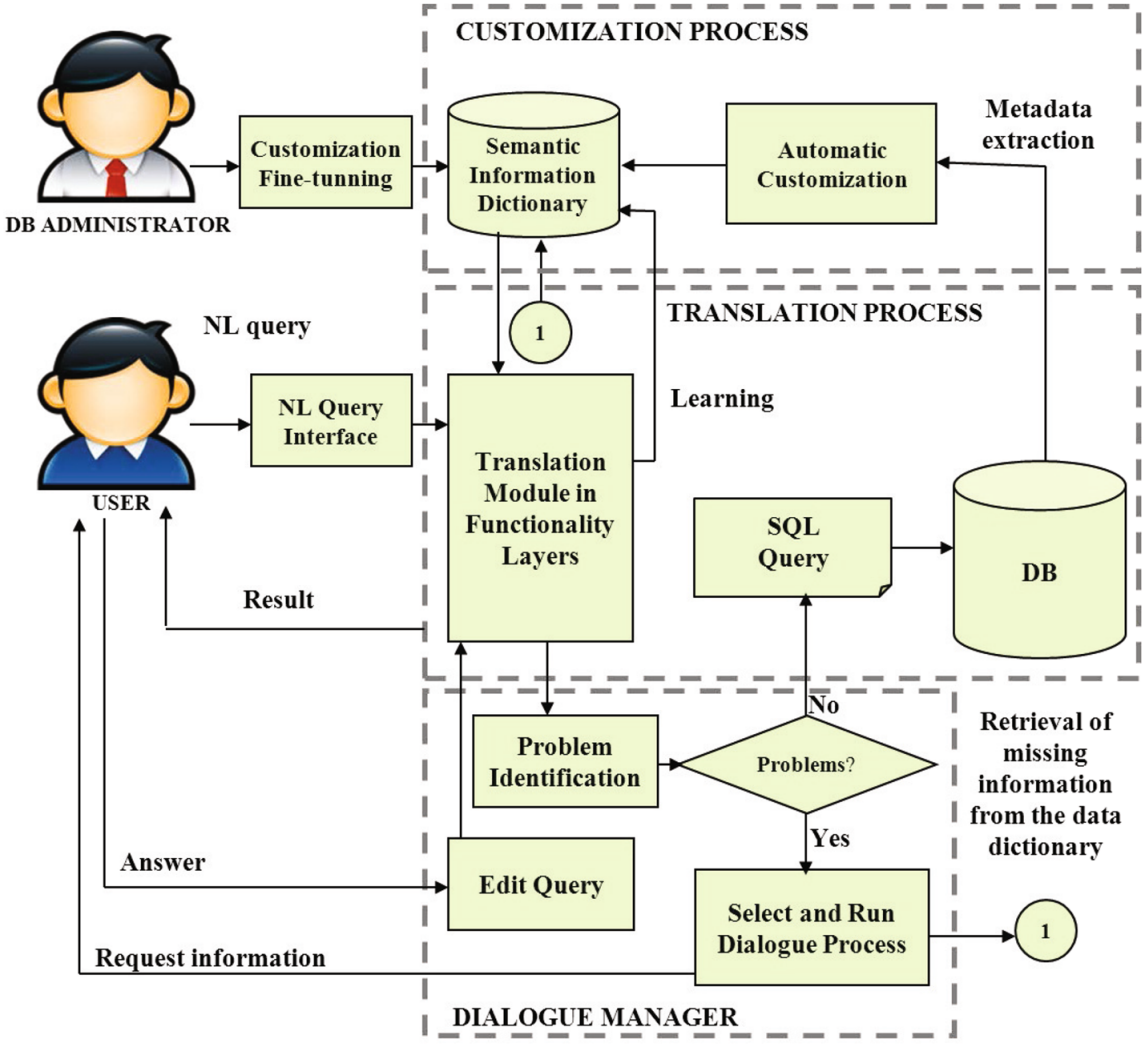
Proposed architecture for the NLIDB

**Fig. 4:**
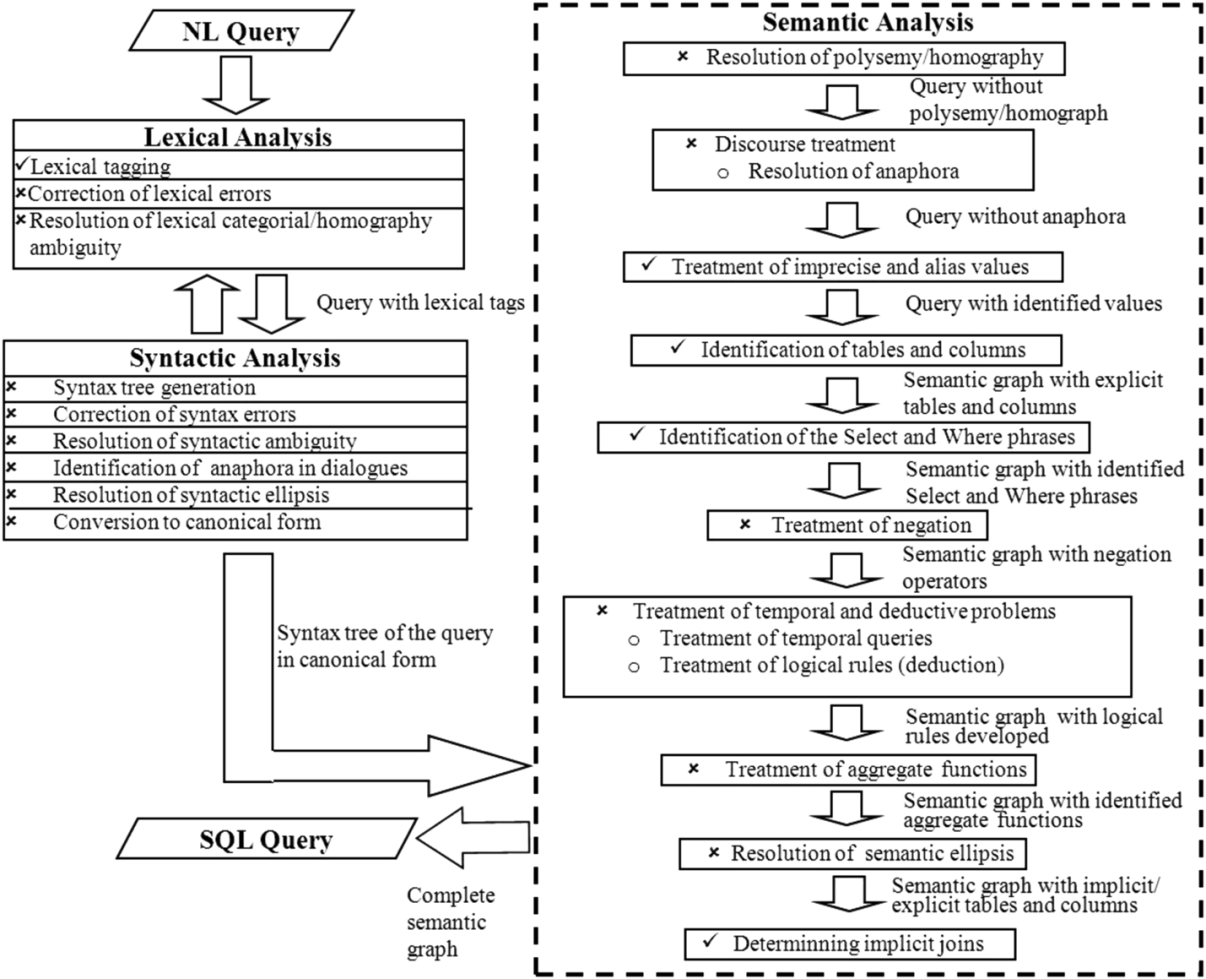
Functionality layers of the translation module

Systems whose design is based on functionality layers (like the OSI model for communication networks) provide the flexibility and modularity for implementing more complex processing strategies than systems designed otherwise. Figure 3 depicts the general architecture of our NLIDB, and Fig. 4 details the architecture of the most important part of the interface: the translation module, whose design is based on functionality layers.

The functionality layers described in this subsection were devised for providing a desirable functionality in a NLIDB; i.e., each layer deals with a problem that must be solved so our interface can obtain a correct query translation; for example: treatment of imprecise and alias values, resolution of semantic ellipsis. It is important to mention that the functionality layers included in our architecture are those that we think any NLIDB should have in order to solve most of the problems found in the translation process from natural language to SQL. The layers that have been implemented so far are indicated by a check mark, while those that are planned for future work are indicated by a cross. A brief explanation of the functionality layers of the translation module is given in the rest of this subsection.

#### Lexical analysis

 It carries out a lexical tagging process by retrieving the syntactic category of each query word from a lexicon stored in a database. In this layer, lexical errors should be corrected and syntactic category ambiguity and homography problems should be resolved. The result obtained consists of the tagged query.

Algorithm 1 shows the pseudocode that describes the general structure of the lexical analysis implemented. In the pseudocode, *Q* is the query introduced by the user, $Q_i$ is a token of query *Q*, *n* is the total number of tokens in *Q*, and *L* is a temporal list used to store temporally grammatical categories^1^ of token $Q_i$. At line 4, for each token of query *Q*, a list of possible grammatical categories of $Q_i$ are stored in *L*; in lines 6 to 9, if the list *L* is not empty, all the grammatical categories found in the token are assigned to $Q_i$, otherwise, the token $Q_i$ is tagged as a possible search value. 
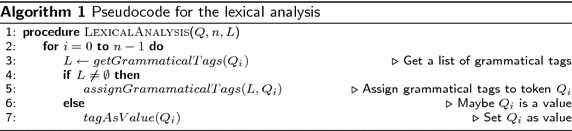

#### Syntactic analysis

Using the tagged query, a syntax tree of the query is built, where syntactic errors have been corrected, syntactic ellipsis has been resolved, and anaphora problems are detected. Since this layer has not been implemented, a shallow analysis is performed instead, which is explained in “Processing of queries” section.

#### Semantic analysis

From the tagged query, a representation of its meaning is constructed, which can be used for translating it to SQL. This layer is the most complex, since most of the problems are related to understanding the meaning of the query. This layer is constituted by the following sub-layers:

1. *Resolution of polysemy and homography. *The polysemy and homography problems detected in the lexical analysis layer are resolved in this sub-layer.
2. *Discourse treatment.* The process performed in this sub-layer resolves the anaphora problems detected in the syntactic analysis.
3. *Treatment of imprecise and alias values.* In this sub-layer words that denote imprecise values (i.e., words that represent value ranges) and aliases (i.e., words for referring to numerical values, such as noon, dozen, third) are detected and dealt with. Algorithms 2 and 3 show the pseudocodes (as implemented) that describe the general structure of this functionality sub-layer.
  The pseudocode of Algorithm 2 describes the process of tagging imprecise values. The process begins at line 1, for each token of query *Q*; if $Q_i$ is an imprecise value (found in the SID), the DB column, lower bound and upper bound specified in the SID for the imprecise value are associated to $Q_i$ (lines 2 to 7). 
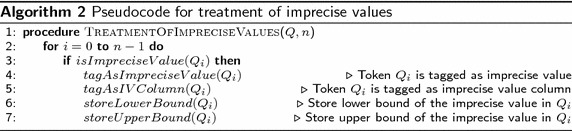
  Algorithm 3 describes the process of tagging alias values. The process begins at line 1, for each token of query *Q*; if $Q_i$ is an alias value (found in the SID), the equivalent value (specified in the SID) for the alias value is associated to $Q_i$ (lines 2 to 5). 
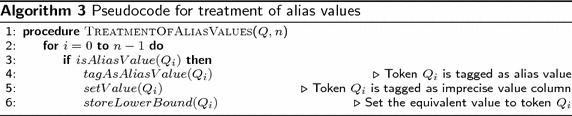
4. *Identification of tables and columns.* Once the search values are identified in the NL query and the tokens have been tagged, the task of this sub-layer is to identify the DB tables and columns referred to by query words and phrases, which can be nominal, verbal, adjectival, or prepositional.
  Algorithm 4 shows the pseudocode (as implemented) that describes the general structure of this functionality sub-layer. *S* is a temporary string to store tokens that form a phrase and *j* is the position of the last token of $Q_i$ that constitutes the phrase. The process begins at line 1. For each token of query *Q* that constitutes a phrase, the token is stored in *S*. Lines 4 to 6 verify if *S* is a grammatical descriptor that describes a column in the SID; if so, all the tokens from *i* to *j* are tagged with the column name, and all the tokens are marked as elements of the phrase. If *S* is not a descriptor for a column, in lines 8 to 11, it is determined if *S* is a grammatical descriptor that describes a table in the SID; if so, all the tokens from *i* to *j* are tagged with the table name, and all the tokens are marked as elements of the phrase.
  At this point, it is worth pointing out that, unlike other NLIDBs (such as ELF and C-Phrase), the translation process of our NLIDB does not look into the database nor the data dictionary for search values in order to determine the DB columns involved in the SQL query. We avoid doing this, because it is impractical for large databases (databases whose tables have more than 100,000 rows), as the experiment described in Pazos et al. (2014) for ELF shows.
5. *Identification of the Select and Where phrases.* From the identification of tables, columns and search values, a heuristic method is used to determine the segments of the query that constitute the Select and Where phrases; where the Select phrase and the Where phrase are the query segments that will be respectively translated to the Select clause and the Where clause of the SQL statement. The result of this sub-layer is the association of search values to columns and the determination of the columns whose data the user wishes to de displayed.
  Algorithm 4 shows the pseudocode (as implemented) that describes the structure of the Identification of the Select and Where phrases. *L* is a temporary list to store the position of the tokens that are values, *j* is the position of the value selected, and *n* is the number of values in query *Q*. At line 1, first the position of the search values are determined and are stored in list *L*. The process begins at line 2, for each search value of query *Q* from the last element to the first of the list. If the token to the left of the search value refers to a column, token $Q_j$ and the previous token are marked as part of the Where phrase (lines 3 and 4). Otherwise, if the token to the left of the search value refers to a table, token $Q_j$ and the previous token are marked as part of the Where phrase (which is an error, but it is tagged with the purpose of identifying the type of error) (lines 6 and 7). Otherwise, if token $Q_i$ is part of a set of search values (e.g., flight number 1, 2, 3 and 4), token $Q_j$ and the previous tokens are marked as part of the Where phrase (lines 9 and 10). Otherwise, if token $Q_i$ is part of a Between phrase (i.e. departure time between 1200 and 1900 h), token $Q_j$ and the previous tokens are marked as part of the Where phrase (lines 12 and 13). Otherwise, continue with the next token value in the list. Finally, in lines 21 and 22, if there are tokens not tagged as Where phrase, all the remaining tokens are tagged as part of the Select phrase. 
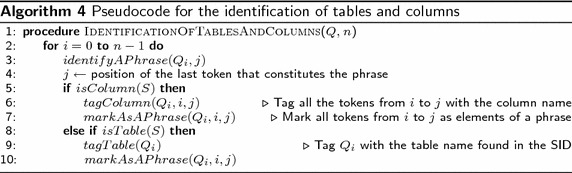
6. *Treatment of negation.* This sub-layer identifies and deals with the lexical components of the query that denote negation. Queries that involve negation can be easily expressed in natural language; for example: *which are the golf courses without practice course?* or *which camping grounds do not require a camping permit?* However, they might pose some difficulty when they are interpreted for translation into SQL: some translation approaches for NLIDBs do not distinguish between negative and affirmative sentences, for example, those described in Androutsopoulos et al. (1993), Minock (2010), Popescu et al. (2004a), Stratica et al. (2005).
7. *Treatment of temporal and deductive problems. *Temporal queries are issued to a database that permits storing the history of values that a piece of data may adopt over time. Temporal databases have a temporal data model and a temporal version of SQL. An example of a temporal query is the following: *who was the spokesman of the Soviet Embassy in Baghdad during the invasion of Kuwait? *Additionally, deductive queries are those that are based on deductions by using inference. This type of queries are usually formulated to deductive databases, which may be implemented on relational databases but include facts and logic rules. An example of a logic rule is: every father of a father is a grandfather; therefore, assuming there is no information on grandfathers in a database, the query *who is the grandfather of John?* based on the preceding rule must return the desired result.
8. *Analysis of aggregate functions and grouping. *This sub-layer identifies and deals with the lexical components of the query referring to aggregate functions and grouping (Group By); for example words such as average, how many, minimal, maximal, smallest, largest, first, best, for each, etc. are used to refer to aggregate functions.
9. *Resolution of semantic ellipsis. *The term semantic ellipsis refers to the omission of important words in the wording of a query written in natural language; more specifically, the omission of words for specifying DB tables or columns involved in the query, which the user assumes they can be guessed. The task of this sub-layer is to detect several ellipsis problems that were classified according to a typification described in Pazos et al. (2010). These problems are solved by an interaction with the user employing clarifying dialogues (Dialogue Manager in Figure 1).
10. *Determining implicit joins.* Once all the tables and columns referred to in the query have been identified, sometimes the query can not be translated to SQL because the graph that represents these tables and their relations is not connected. In this case it might be necessary to include additional tables (not mentioned in the natural language query) and arcs (relations between tables) in order to generate a connected graph. For overcoming this problem this sub-layer uses a heuristic method, which consists of performing a breadth-first traversal of the semantic graph that represents all of the DB tables and relations between tables in order to find the smallest set of tables for generating a connected sub-graph than includes all the previously identified tables. Finally, from this resulting sub-graph the SQL statement can be easily generated. 
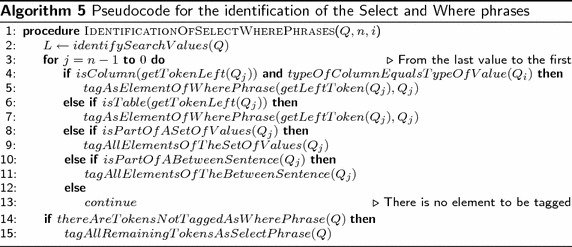
  Algorithm 6 shows the pseudocode (as implemented) that describes the functionality layer for determining implicit joins. In the pseudocode, *G* is the semantic graph that contains the relationships between tables (this information is obtained from the SID), *T* is a list that stores the tables involved in the query. First at line 1, the semantic graph is loaded from the SID into *G*. Then, in line 2, the tables involved in *Q* are stored in *T*. In lines 3 and 4, if the number of tables involved is just one, it returns the table because there is no need to create a search tree. Otherwise, if the number of tables involved in *Q* is larger than one, for each pair of tables in *T* a breadth first search tree is created starting from one of the tables evaluated, if there is a path between the tables being evaluated, then return the foreign keys of the path found between the two tables evaluated; otherwise, return an error because there is no path between the tables evaluated. 
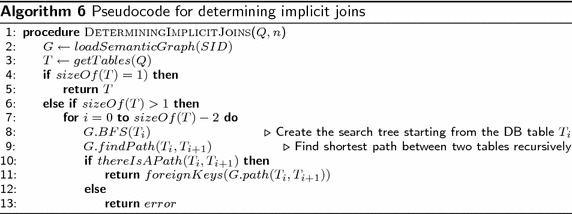

For better understanding the process performed by our NLIDB, an example of the process for a query with several of the aforementioned problems is explained in “Example of the application of the semantic information dictionary” section.

### Processing of queries

The interface that we are currently developing is part of a far-reaching project aiming at solving most of the problems occurring in queries, described in Pazos et al. (2013) and dealt with by the architecture depicted in Fig. 4. The version presented in this article deals only with the problems implemented so far; i.e., those indicated by a check mark in Fig. 4.

For exemplifying the translation process, the following NL query will be used:

List round trip fares from Philly to DFW arriving in the evening.

**Fig. 5:**
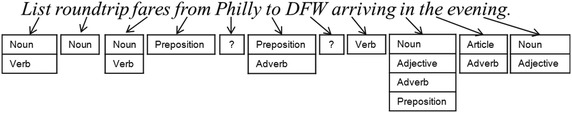
Example of lexical tagging

The translation process starts by lexically tagging each token of the query. For each token its tag consists of its syntactic category (or categories) as shown in Fig. 5. The tagging is performed by looking for the token in a lexicon that contains all the language words (approximately 1,132,000 words). If a token can not be found in the lexicon, it is tagged with a question mark, and it is a candidate for being considered as a search value. It is important to make clear that our NLIDB only answers queries in Spanish; however, because of the similarities among European languages, the process of our NLIDB can be applied also to English. Therefore, for the sake of clarity the examples presented in this article are for English.

**Fig. 6:**
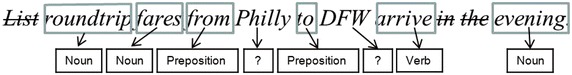
Example of shallow analysis for discriminating syntactic categories

Next, instead of the syntactic analysis mentioned in Fig. 4, a shallow analysis is applied to the query in order to obtain only one syntactic category for those words that have two or more, as shown in Fig. 6. The shallow analysis consists of some heuristic rules. Additionally, irrelevant words are ignored; for example, list (a verb of an imperative sentence), the (article) and in (a preposition is usually ignored, unless an entry of the SID indicates that it is used for referring to a column, such as preposition from).

**Fig. 7:**
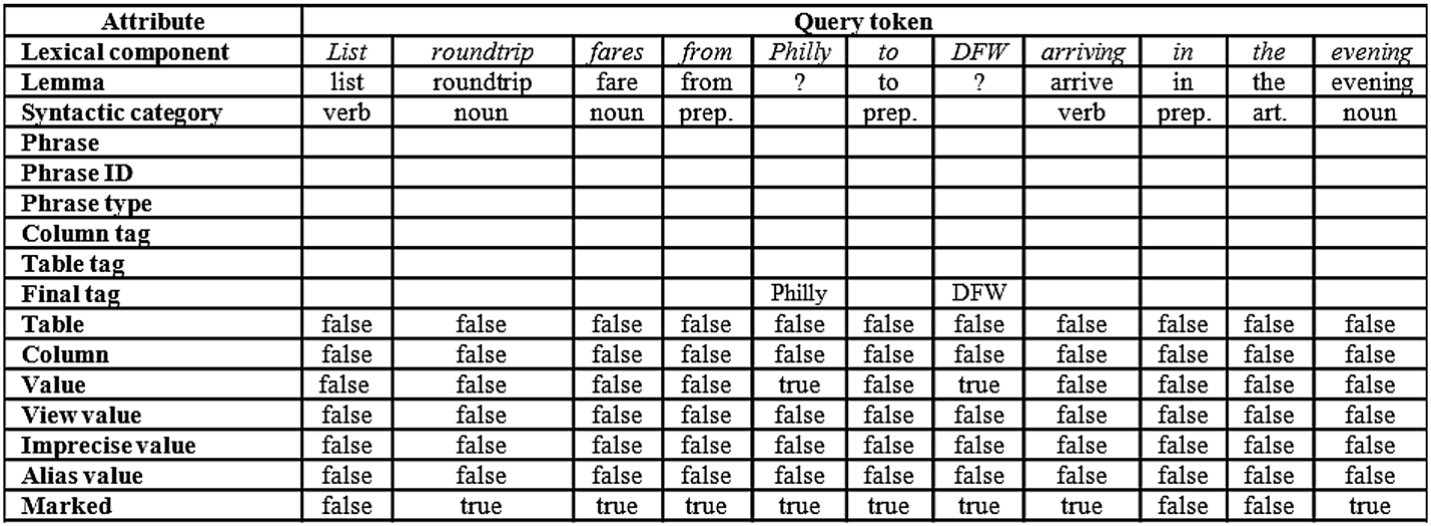
Example of information generated during the lexical analysis

For detailing the processing of a query, Fig. 7 depicts a table that shows the information generated for each query token. A token can be treated as an independent element, which is a structure that contains a set of attributes that are used for processing the query. The attributes contained in each structure of the token are: lexical component, lemma, syntactic category, phrase (sequence of words that contains the token), phrase identifier, type of the phrase (Select or Where) it is part of, tag(s) of the column(s) referred to, tag(s) of the table(s) referred to, final tag, indicator if it refers to a table, indicator if it refers to a column, indicator if it is a search value, indicator if it is a search value of a view, indicator if it is an imprecise value, indicator if it is an alias value, and indicator if it is marked for being processed.

The rows with headings *Lexical component*,* Lemma*, *Syntactic category*,* Final tag*, *Value*, and *Marked* (Fig. 7) show the information generated by the lexical analysis for the tokens of the NL query. It is convenient to make clear that the value *Philly* in the *Final tag* row is not yet the final value for the token *Philly*, since this is an alias value that has to be converted to its equivalent value. The values *false* in the *Marked* row indicate that the corresponding tokens are irrelevant for the translation.

**Fig. 8:**
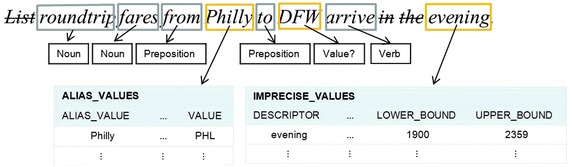
Example of treatment of imprecise and alias values using the SID

The first sub-layer of the semantic analysis that has been implemented deals with the treatment of imprecise and alias values (Fig. 4). The corresponding process looks for alias values and imprecise values that are declared in the SID; for example, the token *Philly* has been declared for referring to the search value *PHL*, and the token *evening* has been declared for referring to the value range 1900–2359 (Fig. 8). The rest of the tokens that have not been identified so far, are considered search values since they are not language words, *DFW* in the example. The rows with headings *Final tag*, *Value*, *Imprecise value* and *Alias value* (Fig. 11) show the information generated by the treatment of imprecise and alias values.

**Fig. 9:**
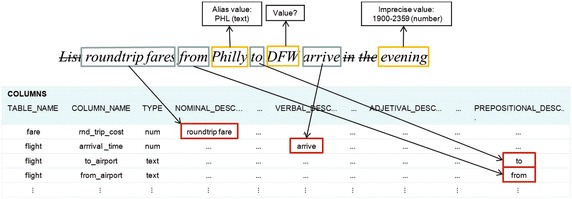
Example of table and column identification using the SID

The next sub-layer deals with the identification of DB tables and columns. In this sub-layer a search in the SID of the tagged tokens of the query is carried out, either as individual words or phrases of different syntactic categories (nominal, verbal, adjectival, or prepositional). If there is a word/phrase in the SID that matches a word/phrase of the query, then the query word/phrase is tagged with the table or column referred to by the word/phrase. As shown in Fig. 9, according to the semantic information stored in the SID, the nominal phrase *round trip fare* refers to column *rnd_trip_cost* of table *fare*, the verb *arrive* refers to column *arrival_time* of table *flight*, and the prepositions *from* and *to* refer to columns *from_airport* and *to_airport* of table *flight*.

The rows with headings *Phrase*, *Phrase ID*, *Column tag* and *Table tag* of the table depicted in Fig. 11 show the information generated by the identification of DB tables and columns. Notice that the third column of the table results from the combination of the third and fourth columns of the table depicted in Fig. 7, which occurred because the phrase *round trip fare* was found in the SID as descriptor of column *fare.rnd_trip_cost*. The list 1, 2 in the *Phrase ID* row indicates that the phrase is constituted by tokens 1 and 2 (note: the numbering of tokens starts at 0).

**Fig. 10:**
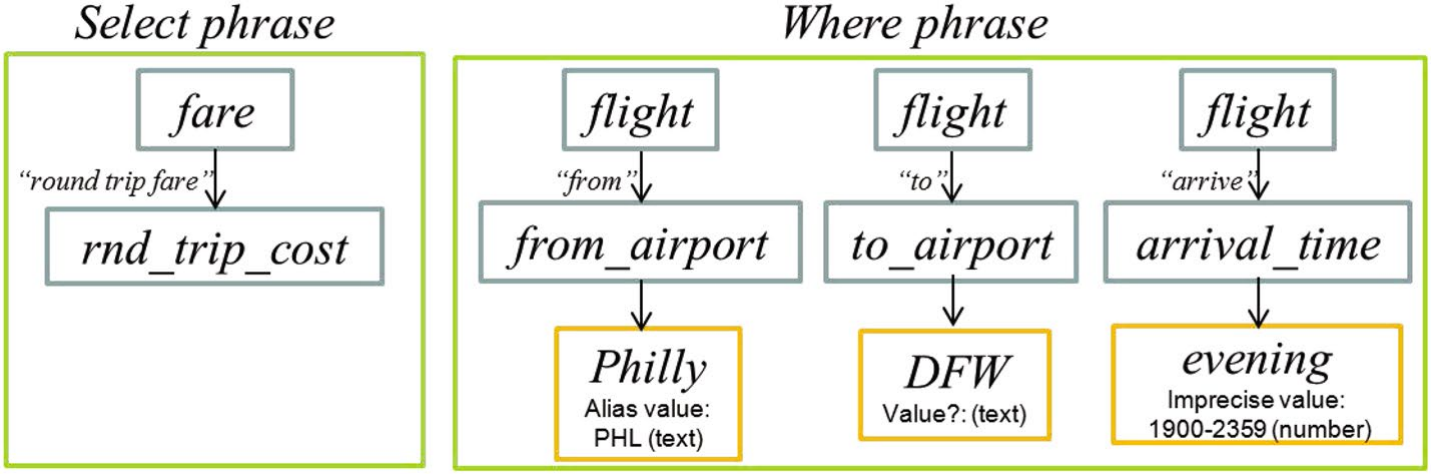
Example of separation of the Select and Where phrases

**Fig. 11:**
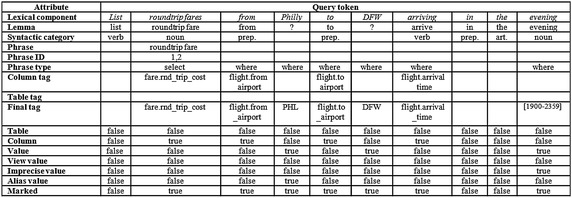
Example of information generated during the semantic analysis

The next sub-layer deals with the identification of the Select and Where phrases. After the tables and columns have been identified, as well as the search values, now it is possible to divide the query into its Select and Where phrases. For performing this task each search value present in the query is associated to the column on its left, as shown in Fig. 10. The set of pairs of column-search values constitute the Where phrase. The rest of the column-tagged tokens are considered as constituents of the Select phrase. The row with heading *Phrase type* (Fig. 11) shows the information generated by the identification of the Select and Where phrases.

**Fig. 12:**
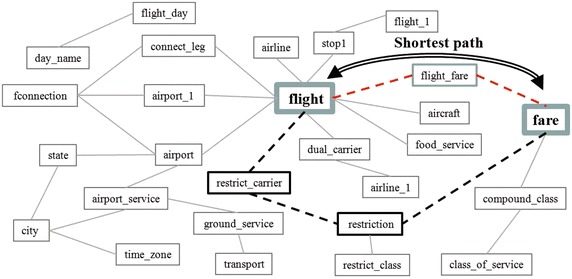
Example of determination of implicit joins

**Fig. 13:**
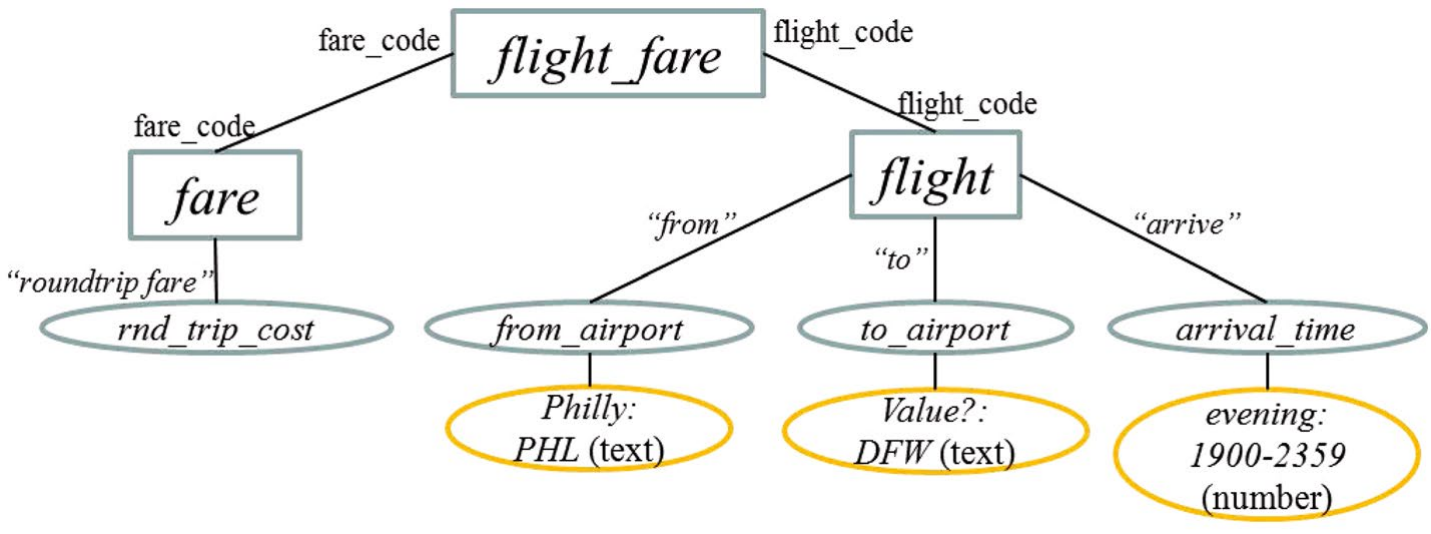
Example of connected semantic graph

The next (and last) sub-layer implemented is in charge of determining implicit joins in the query when necessary. For carrying out the generation of an SQL statement it is necessary that the graph constituted by the tables identified and the join conditions (search conditions that involve one column from one table and one column from another table) be a connected graph, like the one depicted in Fig. 13. When this is not the case, then it is necessary to include tables and join conditions for obtaining a connected graph as explained in “Proposed architecture” section. For this example, Fig. 12 depicts a fragment of the ATIS database schema that shows tables *fare* and *flight* and some adjacent tables (i.e., tables related by a foreign key), as well as segmented lines that denote foreign-key relations between tables. At this point of the process, the semantic graph of the query is unconnected and consists of two components: one includes table *fare* and the other includes table *flight*. For this example, our heuristic algorithm finds the shortest path between tables *fare* and *flight* (Fig. 12), which indicates that it is necessary to include table *flight_fare*, and the corresponding join conditions *fare.fare_code = flight_fare.fare_code and flight.flight_code = flight_fare.flight_code* (Fig. 13).

The final result of the query processing consists of a semantic graph that contains all the information that the NLIDB needs for “understanding” the query (Fig. 13). From the semantic graph, the process for obtaining the SQL statement is straightforward.

Additional details on query processing can be found in Aguirre (2014).

### Example of the application of the semantic information dictionary

As previously mentioned, the SID of our NLIDB permits using words or phrases of syntactic categories such as nouns, verbs, adjectives and prepositions for relating them to DB tables or columns. In addition, it permits storing another type of information related to values (i.e., data that may be stored in the database), such as imprecise values and alias values. For exemplifying the advantages of the design of our SID, based on the SEDBM model, an example is described next.

#### Use of words or phrases of different syntactic categories

Usually DB tables and columns are referred to by nominal words or phrases; however, a study of several query corpora revealed that words and phrases of verbal, adjectival and prepositional categories are also used in natural language queries for referring to tables and columns (Pazos et al. 2005). For illustrating this case, let us consider the following query of the well known ATIS database: *Give me a list of flights from DFW to BOS that arrive before 700*.

**Fig. 14:**
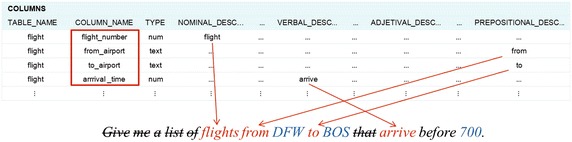
Example of grammatical descriptors in column descriptions

The semantic analysis of this query shows that the words *flights*, *from*, *to* and *arrive* refer to columns of table *flight*. Initially the system tags with syntactic categories each word found in the query. Afterwards, the query is analyzed, and at different moments of this process (Algorithm 4), the system accesses the SID to find out if these words or phrases are present in the grammatical descriptor of some DB table or column. Figure 14 shows how the columns referred to by the abovementioned words are identified in the SID by the column descriptors.

### Customization of the semantic information dictionary

The customization of our NLIDB for a particular database consists of populating the SID with relevant semantic information of the database. The customization is carried out in two steps: automatic customization and fine-tuning.

The automatic customization extracts information from the DB schema and populates the SID by associating the descriptions stored in the schema to DB tables, columns and relations between tables; for example, the description for column *flight.from_airport* in the schema is *origin airport*, so this phrase is stored in the SID as a descriptor for the column. Specifically, for each DB table it extracts: DB name, table name, and a nominal descriptor (most DB management systems permit storing a description for tables); for each DB column it extracts: DB name, table name, column name, data type, an indication if the column participates in the primary key, and a nominal descriptor; and for each relation between tables it extracts: DB name, column name of the table referred, name of the table referred, column name of the referring table, and name of the referring table.

The fine-tuning of our NLIDB is carried out as follows: first the customizer has to detect a query that has not been correctly translated by the interface, and then he/she has to determine what information is lacking (or is wrong) in the SID that could be causing the error. To this end, the user has to compare the SQL statement generated by the NLIDB and the correct SQL statement, and he/she has to detect the differences; afterwards, by taking into account the NL query and the database schema, the user has to find out what information he/she has to store (or correct) in the SID. To this end, our NLIDB has a domain editor which allows associating words/phrases of different grammatical categories (nominal, verbal, adjectival and prepositional) to DB tables and columns. For example, the NLIDB will not be able to interpret the query *How much is a round-trip fare from BOS to DFW?*, because *BOS* is a value stored in column *flight.from_airport*, whose descriptor is *origin airport *(as mentioned before). This problem can be solved by using the domain editor to include the word *from* as another descriptor for column *flight.from_airport*. Other elements that can be defined using the editor are imprecise values and alias values.

For example, after the automatic customization of the NLIDB, the following query How much is a round trip fare from BOS to DFW? is incorrectly translated to

SELECT fare.rnd_trip_cost FROM fare;

however, the correct SQL statement is

SELECT fare.rnd_trip_cost

FROM fare, flight, flight_fare

WHERE flight.from_airport LIKE ‘BOS’ AND flight.to_airport LIKE ‘DFW’

AND flight.flight_code = flight_fare.flight_code

AND flight_fare.fare_code = fare.fare_code.

By looking at the two SQL statements, it is clear that the interface does not “understand” the phrase *from BOS to DFW*; this is so because, after the initial customization, prepositions *from* and *to* are not stored in the SID. Incidentally, the SID only has the following nominal descriptor for column *flight.from_airport*: origin airport, and similarly for column *flight.to_airport*. In order to correct this situation, it is necessary to perform a fine-tuning of the interface, which consists of storing preposition from as a prepositional descriptor for column *flight.from_airport* (Fig. 2) and storing preposition to as prepositional descriptor for column *flight.to_airport*. After this process the interface will “understand” that preposition from followed by a search value *X* is used for referring to column *flight.from_airport* and likewise for preposition *to*.

In general, once the NLIDB has been customized for “understanding” certain words/phrases, then it will correctly interpret them when they are found in previously unseen queries. For example, the query *List all flights from DEN to PIT* and *list the fares* is not correctly answered after the automatic customization; therefore, it is necessary to fine-tune the interface by storing: the nominal descriptor *flight* for column *flight.flight_number*, the prepositional descriptor from for column *flight.from_airport*, the prepositional descriptor *to* for column *flight.to_airport*, and the nominal descriptor *fare* for columns *fare.one_way_cost* and *fare.rnd_trip_cost*. After this process, the interface will correctly answer this query.

Additionally, the query *Please show me flights that leave after noon* is not correctly translated after the automatic customization; therefore, it is necessary to fine-tune the interface by storing: the verbal descriptor *leave* for column *flight.departure_time* and an alias value for *noon*, which has to be declared to represent 1200. In this case, the interface already “understands” that *flight* refers to column *flight.flight_number*. After this process, the interface will correctly answer the last query.

Finally, if the interface is given a previously unseen query such as *List fares for all flights leaving after noon from BOS to BWI*, it will correctly translate it, since it already “understands” the meaning of *fare*, *flight*, *leave*, *noon*, *from* and *to*. However, with this customization information, the current version of the interface would not be able to answer the query* List fares for all flights departing after noon from BOS to BWI*, because it does not “understand” the meaning of *departing*. The new version under development includes a synonym dictionary that will permit answering this query; however, it would not correctly answer the query *List fares for all flights flying after noon from BOS to BWI* because, in general *fly* is not considered synonym of *leave*.

The customization of C-Phrase is carried out in two phases. In the first one, the DB administrator manually generates a file that contains information on the connection to the DB and the data dictionary. The file defines the dictionary as an ontology that describes the relations between tables/columns and words that can be used in queries to refer to them, it also contains match patterns for each table/column, which define the phrases that are expected to be used for referring to the table/column; for example, table *state* can be referred to by the word *state* and has 38 patterns defined for it. An example of this pattern is *$C5 most populated* is interpreted by the interface as *X|STATE(X) and TOP-POPULATION ($C5,X)*, where *X* and *$C5* denote a state name (or set of state names to show) and a numeric variable that indicates the number of states to show. Additionally, the file defines text substitutions for words/phrases that may occur in queries; for example, the file defines a substitution for *how many square kilometers*, which if found in a query it would be replaced by *sum area*. The customization supplied by the implementers for Geobase has 75 substitutions. It is important to point out that the customization file provided by the implementers for Geobase has 1593 lines of text (C-Phrase 2010).

The second phase is performed for fine-tuning the data dictionary and adding/modifying substitutions. C-Phrase has an authoring tool that allows editing the names of DB tables/columns, associating patterns to tables/columns, and editing substitutions. For example, the pattern *containing fewer that $C1 people* is interpreted by the interface as *X|STATE(X) and X.POPULATION < $C1*, (where *X* and *$C1* represent a state name and a search value). In this process the DB administrator has to define the last expression and one or more patterns for it.

The customization of ELF is performed in two phases also: the first phase is automatic and the second is manual. In the first phase, ELF stores in the data dictionary the names of tables/columns extracted from the DB schema and assigns *synonyms* to tables/columns, which are words that are derived from their names. ELF also stores in the data dictionary the values stored in the database: for each value it records the name of the column where it is stored. This information is used by ELF to identify the tokens in the NL query that are search values and to find out which column or columns store the identified search value.

The second phase is executed for fine-tuning the data dictionary. The ELF editing tool allows looking for a table or column in order to add/delete synonyms for it; for example, a new synonym *class* could be added for column *SupplierID*. The Phrase Editor allows defining substitutions for words or phrases, like C-Phrase.

The main aspects of our approach that differ from those of ELF and C-Phrase are the following:

- No use of patterns for query translation. C-Phrase associates patterns and rules for performing a NL query translation. A disadvantage of this approach is that it is necessary to define a new pattern when a user formulates a NL query not considered in the set of patterns. Our approach is more flexible, since our NLIDB only stores descriptors in the SID for tables and columns, which are used by a semantic analysis process that determines, from the NL query, the information of tables, columns and search values required to construct the SQL statement.
- No substitution of query text. Unlike ELF and C-Phrase, our NLIDB does not use text substitution. A disadvantage of text substitution is that it may yield perplexing results. For example, for the following query from the Geobase corpus: *what is the shortest river in the usa?*, C-Phrase responds *there is no river with name “rivera”*. The explanation for this result is that the customization provided by the implementers specifies that the string *“ in the us”* has to be replaced by an empty string (C-Phrase 2010); therefore, the query is internally rephrased as follows:* what is the shortest rivera*, but *rivera* is not a value stored in the database.
- No storage of DB data in the data dictionary. As mentioned before, ELF stores DB data in its data dictionary, which limits its ability to query large databases: its dictionary may become as large as the database to be queried. Additionally, ELF relies very much on this information for query translation and sometimes is misled by it; for example, ELF translates the query *Which states does the Mississippi river run through?* into *SELECT DISTINCT HighLow.state_name FROM HighLow WHERE (HighLow.lowest_point = “mississippi river”)*. The explanation for this result is that it finds the value *mississippi river* in table *HighLow*. Furthermore, this approach works fine for static databases, but it fails when databases undergo changes (insertions, deletions and updates), because ELF might not be able to identify search values and the corresponding columns.
- No searching of values in the database for query translation. C-Phrase searches the database for values in order to determine the column where the value is stored. For example, for the query *How big is mexico*, C-Phrase responds *There is no lake with name “mexico”*, *There is no mountain with name “mexico”*, *There is no state named “mexico”*, *There is no river with name “mexico”*, *There is no mountain with state “mexico”*, and *There is no city named “mexico”*. The disadvantage of searching a value in the database for determining the column that stores the value, is that it may be prohibitive for large databases.

## Experimental evaluation

Comparative evaluation of the performance of NLIDBs is a difficult task, since there is no established benchmark that permits comparing the performance results from different NLIDBs. This evaluation aims at comparing the customization effectiveness of our NLIDB with respect to that of ELF. We decided to use ELF for comparison because it is easily available and it is one of the few surviving commercial NLIDBs.

In this experiment the interfaces are tested using a query corpus for the ATIS database, which deals with information on airline flights. We decided to use the ATIS database for testing because it is an example of complex medium-size databases that can be found in real-life applications. Because of the complexity of the benchmark (28 tables, 127 columns, and a corpus with 85 % of elliptical queries), only a few NLIDBs have dared to evaluate their performance against this benchmark (Table 1).

For the experiments a subset of the ATIS corpus was extracted, which consists of 70 significant queries (i.e., excluding similar queries) that involve problems of the following types: use of different syntactic categories (nouns, verbs, adjectives and prepositions), use of imprecise and alias values.

At this point it is important to mention that the current version of our NLIDB is under development; therefore, it was tested with neither dialogue manager nor a learning module. Thus, our interface and ELF were tested in similar conditions.

In each of the experiments described next, the customization of the interfaces was performed in two steps: first an automatic customization was carried out (both interfaces have this functionality), and finally the initial customization was manually fine-tuned. The performance results were evaluated using the recall metric, described in Pazos et al. (2013), Lu (2013):

1 $$\begin{aligned} recall=\frac{total\ number\ of\ correct\ queries}{total\ number\ of\ queries}\times 100 \end{aligned}$$

As shown by expression (1), recall is the percentage of correctly answered queries with respect to the number of queries input to the interface.

The first experiment was conducted using the 70-query corpus mentioned before, and the customization was performed by a group of 28 undergraduate students majoring in engineering in computer science, which is the minimal academic background required for customizing a NLIDB. In order to avoid bias towards any of the interfaces (ELF and ours), the group was divided into two subgroups, each with similar average grades. Each subgroup customized one of the interfaces.

For testing the easiness of the interface customization process, the students were given 2 h. Since fine-tuning the customization of ELF is particularly obscure, the students received two manuals (one for each interface) about 10 pages long several days in advance, so they could read them prior to the test. Both manuals included the same examples showing how to customize the interface for correcting some translation errors. Additionally, together with the manuals, the students were given an 11-page document with a description of the ATIS database, which includes a brief description of each table and column, as well as the list of the 70 queries of the corpus (both in English and Spanish) together with the translation of each query into SQL, so the students could receive feedback on the quality of their customization.

The average results obtained in the first experiment are summarized in Table 2. Our NLIDB obtained 44.69 % recall, while ELF obtained 11.83 % recall. It is convenient to mention that one student could make our NLIDB achieve 90 % recall. The fifth column of Table 2 shows the average time that it took students to fine-tune the interface in order to increase by 1 the number of correctly answered queries. This column shows that it took 94.4 min to fine-tune ELF for increasing by 1 the number of correct queries; while for our NLIDB it took 8.4 min.

**Table 2:** Evaluation results of the first experiment

NLIDB	Total queries	Correct w/initial customization	Correct w/fine-tuning	Minutes for customizing one query	Recall w/initial customization (%)	Recall w/fine-tuning (%)
ELF	70	7	8.28	93.4	10	11.83
Our NLIDB	70	17	31.28	8.4	24.28	44.69

The second experiment was conducted using the same corpus of queries; however in this case, a group of 18 undergraduate students majoring in engineering in computer science. Since this was a smaller group, we used cross-testing in order to avoid bias. To this end, first we divided the group into two subgroups: A and B; then in the first session subgroup A customized our NLIDB and subgroup B customized ELF; finally, in the second session subgroup A customized ELF and subgroup B customized our interface. The results obtained from the second experiments are summarized in Table 3, which shows that our interface achieved 77.05 % recall, and ELF obtained 13.48 % recall. The fifth column of Table 3 shows that it took 49.2 min to fine-tune ELF for increasing by 1 the number of correct queries; while for our NLIDB it took 3.2 min.

**Table 3:** Evaluation results of the second experiment

NLIDB	Total queries	Correct w/initial customization	Correct w/fine-tuning	Minutes for customizing one query	Recall w/initial customization (%)	Recall w/fine-tuning (%)
ELF	70	7	9.44	49.2	10	13.48
Our NLIDB	70	17	53.94	3.2	24.28	77.05

As mentioned in “Introduction” section, the success rate of NLIDBs highly depends on the quality of their customization; moreover, the quality of a customization depends on the ability of the customizer. The large difference in the performances (44.69 and 77.05 % for our NLIDB) reported in Tables 2 and 3 is explained by the fact that the students of the second group are better (as revealed by their academic grades). Notice that the performance for ELF obtained by the second group is also larger than that of the first group. Additionally, the performances within each group also vary widely.

Finally, the following question arises: how far are the recall figures obtained by students from the highest recall that can be obtained from our NLIDB? For answering this question, in the third experiment both interfaces were customized by ourselves. Table 4 shows the results obtained, which shows that our interface obtained 90 % recall. We also include the recall for ELF for comparison. (A demo of our NLIDB is available at: http://nlp.itcm.edu.mx:8080.)

These results show that the performance of our NLIDB when customized by the students is far (a 12.95–44.31 % difference) from the 90 % when customized by the interface implementers. The main reason for this difference is that for customizing the interface it is necessary to have: some understanding of the inner workings of the interface (specifically, the need to associate NL descriptors to DB tables and columns), familiarity with the database schema, and expertise in SQL. Unfortunately, the ability of our undergraduate students is far below that of the implementers. Additionally, the students were given only 2 h for customizing the interface; with more time available they could probably have obtained better results.

Table 4 shows that 10 % (=7) of the queries are not correctly answered by our NLIDB. One of these queries is *Please list only economy class flights leaving after noon*. In this case, the interface fails because *economy* class refers to column *compound_class.economy* (which only stores two possible values *YES* and *NO*) and the search value *YES* for this column is missing in the NL query, though in this context it is implicit. In some real-world databases, there exist columns for storing binary values (YES/NO, True/False, 1/0) that pose this kind of problem.

One of the most difficult situations occurs with the following query: *Please show me the business fare class cost for flight number 1*. The interface can not translate correctly this query because *fare class* is a descriptor for columns *restrict_class.ex_fare_class*, *compound_class.fare_class* and *fare.fare_class*; however, the search value *business* is not a possible value for these columns, but it is a value for column *compound_class.class_type*, whose descriptor is *class type*; therefore, the query could be correctly answered if it were reformulated as follows: *Please show me the cost for business class type for flight number 1*. Unfortunately, it can not be realistically expected that users know this subtlety of the database schema. This problem could be partially overcome if we stored fare class as another descriptor for column *compound_class.class_type*. Unfortunately, this trick does not solve the problem either, because the interface would have to choose one of the four columns referred to by descriptor *fare class*. In this case a clarification dialog interface-user would not be helpful, because it is unlikely that the user knows that *business* is a possible value only for column *compound_class.class_type*.

**Table 4:** Evaluation results with a customization performed by the implementers

NLIDB	Total queries	Correct w/initial customization	Correct w/fine-tuning	Minutes for customizing one query	Recall w/initial customization (%)	Recall w/fine-tuning (%)
ELF	70	7	11	40	10	15.7
Our NLIDB	70	17	63	2.6	24.28	90

A recently developed module of our NLIDB for the treatment of aggregate functions (Fig. 4), allowed to conduct an experiment using the Geoquery250 corpus (Tang and Mooney 2001), which has been used for testing other NLIDBs. In this experiment our NLIDB and ELF were customized by us, and for C-Phrase the customization provided by the implementers was used (C-Phrase 2010). The queries were translated into Spanish, since our NLIDB only answers queries in this language. The experimental results are shown in Table 5. The second column indicates the total number of queries of the corpus, and the third column shows the number of queries that require text substitutions for translation. The fourth column contains the number of correct queries. The fifth column shows the recall obtained considering all the queries (those that require and do not require substitutions), and the sixth column contains the recall excluding the queries that require substitutions.

**Table 5:** Evaluation results using the Geoquery250 corpus

NLIDB	Total queries	Queries with substitutions	Correctly answered	Recall (including substitutions) (%)	Recall (excluding substitutions) (%)
Our NLIDB	250	0	141	56.4	56.4
ELF	250	0	89	35.6	35.6
C-Phrase	250	63 (38 correct)	179	71.6	56.4

The experimental results show that C-Phrase obtains the largest recall (71.6 %) and ELF the smallest (35.6 %), while our NLIDB obtains 56.4 %. Since our NLIDB does not use the substitution mechanism, the last column of the table shows the effectiveness of our translation approach when excluding this mechanism; in this case the recalls for our NLIDB, ELF and C-Phrase are respectively 56.4, 35.6 and 56.4 %. Note: from the 179 correctly answered queries by C-Phrase, 38 required text substitution; thus leaving 141 queries for calculating the recall in the last column.

It is important to mention that this corpus includes 96 queries that involve aggregate functions and grouping; from these, only 37 could be correctly answered by our NLIDB, the other 59 contain different types of problems (e.g., semantic ellipsis, lexical ambiguity, arithmetical operations, deductive queries, non-existent tables or columns) for which the corresponding modules (Fig. 4) have not been developed. The difference in performance of our interface when tested with ATIS (90 % recall) and Geoquery250 (56.4 % recall) is explained by the fact that the ATIS corpus has a smaller percentage of queries not dealt with by the interface.

## Conclusion

NLIDBs are tools that permit users to request information stored in databases more easily than other type of interfaces; i.e., users simply type queries in natural language similarly as they would do when communicating with other people. Unfortunately, despite the large number of NLIDBs developed, there still exist unsolved problems that prevent attaining a successful translation rate that is acceptable for business users (close to 100 %).

Despite the existence of many NLIDBs that claim to be domain independent, porting them to a different database is a complex task, due to the amount of time and tediousness that their customization usually involves. It has been observed that for a NLIDB to obtain good results in the translation process, besides the adequate treatment of the problems occurring in queries, it should provide facilities for customizing adequately the interface, since the more correct information it has the better the identification of elements in the query would be.

We propose the use of the SEDBM model, which includes information needed for solving most of the problems occurring in several query corpora that we analyzed. The design of our SID is based on this model, which endows it with enough robustness for obtaining the required information for a successful translation of most queries (90 % recall). Additionally, the design of the SID makes easier the customization of our NLIDB, when compared with other interfaces such as ELF, as revealed by the experiments described in “Experimental evaluation” section.

For evaluating the performance and easiness of customization of our NLIDB, we chose to compare it with the commercial interface ELF, because it is readily available and is reputed as being one of the best interfaces still on sale. For the comparative experiments we decided to use undergraduate students majoring in engineering in computer science; since we think that, for NLIDBs to be widely used in businesses, it is necessary that they be customized so easily that any professional of this academic level should be able to do it.

The experiments were carried out on the ATIS database, because it is representative of complex databases that can be found in real-life applications. For the experiments we used 70 difficult queries from the ATIS corpus, which involve many problems usually found in queries of different domains. For the performance evaluation, we considered the recall rate (instead of accuracy), since business users are mostly interested in the percentage of correctly answered queries. In many evaluations reported in the literature on NLIDBs, the answer to a NL query is considered correct even if it includes information additional to what it is requested; in this respect, it is important to point out that in our experiments such answers were considered incorrect.

For the experiment that involved the first group of students, our NLIDB obtained a 44.69 % average recall and ELF 11.83 %; while for the experiment involving the second group, our interface attained 77.05 % average recall and ELF 13.48 %. These results lead to the following conclusions: for obtaining a good performance from a NLIDB it is necessary that the interface is correctly customized; moreover, the performance is highly dependent on the ability of the customizer. Our NLIDB is easier to customize than ELF, since for increasing by one the number of correctly answered queries, it takes less time for our interface (3.2–8.4 min, Tables 2 and 3) than what it takes for ELF (49.2–93.4 min). Finally, the explanation of the low performance of ELF, compared with what has been usually reported in the literature (Conlon et al. 2004), is that ATIS is a very complex database and the corpus includes very difficult queries.

The performance of our NLIDB when customized by the students is far (a 12.95–44.31 % difference) from the 90 % when customized by ourselves. At this point it is important to underscore that the students were given only 2 h for carrying out the customization, and it is not clear from the experiments what would the performance be if they had 8 or 16 h. Anyway, the experiment shows that the design of our interface has attained a competitive degree of easiness of customization when compared to a commercial software; however, it is necessary to improve the design so that it permits professionals with undergraduate level to obtain a performance close to 90 %.

Concerning the maximal performance (i.e., when the NLIDB is customized by the implementers), our NLIDB obtained competive results when compared with ELF and C-Phrase, as shown by the results in Table 5.

Though our NLIDB was developed for Spanish, we think that the approach used is general enough to be applied to other European languages such as English, French, Italian, and Portuguese.

Human beings use two methods for dealing with NL sentences: guessing and deep understanding. Small children use guessing because their brains have an incomplete and imprecise model of the world, they do not know the exact meaning(s) of words, and they have not learned the language grammar. In this case they sometimes grasp the correct meaning of a sentence and sometimes do not. Conversely, lawyers can not afford to guess the meaning of law articles, they have to understand their exact meaning in order to determine if an article can be useful for a specific legal issue. In general, human beings use a combination of guessing and understanding. In our NLIDB we are trying to replicate the process performed by lawyers, or more precisely, the process carried out by a DB administrator when understanding a NL query for translating it into SQL.

We have just finished the implementation of a wizard for fine-tuning the interface (Aguirre 2014). Unlike the current version of our NLIDB, this wizard does not require from the customizer to have any knowledge about grammatical concepts (such as nominal phrase, verbal phrase, etc.) nor understanding the inner workings of our interface (i.e., the need for adequately relating query words to DB tables and columns). Preliminary experiments show that the recalls obtained by the NLIDB when fine-tuned with the wizard were 80.53 and 84.82 % for two groups of undergraduate students, which are considerably higher than those obtained without the wizard (Tables 2, 3).

Finally, the use of a semantic information dictionary (based on a new semantically-enriched data model) and a layered architecture for the translation of NL queries to SQL, allows the systematic treatment of problems occurring in NL queries. This approach permits systematically customizing our NLIDB to such an extent that it has made possible to automate its customization by using the wizard.

## Footnotes

1 These grammatical categories are extracted from the lexicon DB.
